# Group size affects spontaneous quantity discrimination performance in wild Western Australian magpies (*Gymnorhina tibicen dorsalis*)

**DOI:** 10.1007/s10071-025-01963-0

**Published:** 2025-05-26

**Authors:** Holly Hunter, Grace Blackburn, Benjamin J. Ashton, Amanda R. Ridley

**Affiliations:** 1 Centre of Evolutionary Biology, School of Biological Sciences, University of Western Australia, Perth, WA Australia; 2 College of Science and Engineering, Flinders University, Adelaide, SA Australia; 3 School of Natural Sciences, Macquarie University, Sydney, NSW Australia

**Keywords:** Spontaneous quantity discrimination, Wildlife, Cognition, Bird, Group size

## Abstract

Animals may benefit from the ability to discriminate between quantities in their environment; for example, when choosing between foraging patches differing in food availability or assessing the size of rival groups. Numerous studies utilising spontaneous quantity discrimination tasks have found that a wide range of species possess the ability to discriminate between quantities, with large interspecific differences being found in these capabilities. However, the *causes* of variation in quantity discrimination have received less attention, particularly when considering intraspecific variation. Here, we use a spontaneous quantity discrimination task to (i) investigate if Western Australian magpies possess quantity discrimination abilities, and (ii) determine the factors that underlie individual variation in this ability. We found that magpies were able to discriminate between two discrete quantities of a food reward and chose the larger quantity of food more often than expected by chance, with their accuracy increasing as the difference between the two quantities of food items increased (i.e. as the ratio decreased). Individual performance on the assay was significantly affected by group size, with individuals from smaller groups choosing the larger quantity of food more often than individuals from larger groups when presented with the 2 vs. 5 combination. This group size difference may arise because individuals from smaller groups benefit more from enhanced quantity discrimination abilities compared to individuals from larger groups due to the greater risk of competition and loss of resources from intergroup conflict with larger groups. Our study is the first to investigate and identify group size as a source of intraspecific variation in spontaneous quantity discrimination abilities and highlights the importance of considering the causes of individual variation in cognitive performance.

**Supplementary Information:**

The online version contains supplementary material available at 10.1007/s10071-025-01963-0.

## Introduction

For many animals, the ability to evaluate and discriminate between quantities (quantity discrimination) plays a fundamental role in everyday decision-making, and is used in many aspects of life (Benson‑Amram et al. 2017; Nieder 2020; Lin et al. 2021). During mating, males of some species may alter their behaviour based on information gained through quantity discrimination. For example, in mealworm beetles (*Tenebrio molitor*), where both sexes mate with many partners, males vary time spent guarding females post-copulation depending on how many rivals the male has faced prior to mating; the more males encountered, the longer he will mate-guard (Carazo et al. 2012). Animals may also use quantity discrimination abilities when faced with predators (Templeton et al. 2005; Gómez‑Laplaza and Gerlai 2011). For example, some songbirds encode information regarding the magnitude of a threat through the number of notes in their alarm or mobbing calls. For black-capped chickadees (*Poecile atricapillus*), two ‘dee’ notes signal a relatively harmless threat, whereas five ‘dee’ notes indicate a predator representing a greater threat, providing conspecifics with more detailed information (Templeton et al. 2005). Moreover, the willingness of individuals to defend against the predator is dependent on the number of individuals calling; the more birds already calling, the more likely others will be to join in (Coomes et al. 2019; Dutour et al. 2021; Dutour and Cordonnier 2023). Quantity discrimination abilities are also likely to be important when intergroup conflict arises, as the ability to evaluate the level of threat based on the size of the rival group may help individuals respond appropriately (Seddon and Tobias 2003; Radford 2003; Benson‑Amram et al. 2011, 2017). For example, male chimpanzees (*Pan troglodytes*) are known to aggressively respond to intruding conspecifics, although their willingness to engage in aggressive altercations depends in part on whether they outnumber the intruders, and more specifically by how much (Wilson et al. 2001; Nieder 2020). Subdesert mesites (*Monias benschi*) display a similar response, where defending groups are less likely to enter into conflict as the number of intruders increases, and even more so as the ratio of males between groups decreases (Seddon and Tobias 2003).

Animals are thought to process numerical stimuli via two complementary systems: (i) the object-file system (OFS; also known as the object-tracking system), which represents small quantities and (ii) the approximate number system (ANS), which estimates larger numerical magnitudes (Hyde 2011; Agrillo 2015; Rugani 2017; Khatiwada and Burmeister 2022). The OFS refers to the automatic and precise storage of a small number of visual objects (Rugani 2017; Nieder 2020; Khatiwada and Burmeister 2022). The OFS allows animals to individuate new objects as they are introduced visually, allocating each one as its own discrete file in the working memory (Rugani 2017). The OFS is defined by the number that can be held concurrently in the working memory, which is typically between one and four (Rugani 2017). The ANS is an inexact system, considered to be used by animals when dealing with larger quantities (Hyde 2011; Khatiwada and Burmeister 2022). The ANS is ratio-dependent and governed by Weber’s Law, in which numerical discrimination increases in accuracy as the difference between two values increases (i.e. as the ratio decreases) (Hyde 2011; Nieder 2020). For instance, combinations with a low ratio, such as 1 vs. 4 (ratio of 0.25), are simpler to discriminate between than combinations with a high ratio, such as 3 vs. 4 (ratio of 0.75). However, whether the ANS and OFS represent distinct methods for processing numerical stimuli or instead operate on a continuum (with the ANS able to operate over the entire numerical range), remains debated (Cordes and Brannon 2009; Hyde 2011; Stancher et al. 2013; Agrillo & Bisazza 2018).

Given its role in many important aspects of life, quantity discrimination has been the subject of a significant amount of empirical attention, and experiments utilising quantity discrimination tasks have reported these abilities across taxa (e.g., fishes, Agrillo et al. 2008; Lucon‑Xiccato and Dadda 2017; Potrich et al. 2022; mammals, Ward and Smuts 2007; Uller and Lewis 2009; Jones and Brannon 2012; Caicoya et al. 2021; Bosshard et al. 2022; birds, Garland et al. 2012; Bogale et al. 2014; amphibians, Uller et al. 2003; Stancher et al. 2015; Khatiwada and Burmeister 2022; reptiles, Szabo et al. 2021, 2023; Lin et al. 2021; Tomonaga et al. 2023; and invertebrates, Howard et al. 2020). To date, however, direct testing of quantity discrimination abilities using cognitive tasks have mostly been confined to captive studies (though see Hunt et al. 2008; Garland et al. 2012). While accurately assessing quantity discrimination in the wild can be challenging due to the potential confounding effects of factors such as predation pressure (Brown and Braithwaite 2005), temperature (Blackburn et al. 2022a; Soravia et al. 2023), hunger or stress (Morand‑Ferron et al. 2016; Shaw and Schmelz 2017; Boogert et al. 2018; Horn et al. 2022), the ecological validity of laboratory testing may be limited (Pritchard et al. 2016; Benson‑Amram et al. 2017). Animals kept in captive environments can be exposed to unique stressors not encountered in the wild, such as being prevented from conducting routine behaviours (Bassett and Buchanan‑Smith 2007), being handled by humans (Hosey 2005), or subjected to social interactions that differ from their usual experience (e.g., in cases where individuals of highly social species are kept in isolation; Arakawa et al. 2008). As such, testing individuals in the wild under ecologically relevant conditions may give a more accurate representation of an individual’s cognitive phenotype.

The causes of variation in quantity discrimination abilities are still relatively unknown (Morand‑Ferron et al. 2016; Bosshard et al. 2022). While many studies have identified that animal species can discriminate between quantities (Hunt et al. 2008; Uller and Lewis 2009; Szabo et al. 2021; Potrich et al. 2022; Khatiwada and Burmeister 2022; Tomonaga et al. 2023), and some have considered causes of interspecific variation (such as diet and sociality) (Bosshard et al. 2022; Szabo et al. 2023), there remains a considerable gap in our knowledge regarding the causes of *intraspecific* variation in quantity discrimination abilities in wild populations (Morand‑Ferron et al. 2016; Lucon-Xiccato and Dadda 2017; Szabo et al. 2022). One factor that may affect quantity discrimination performance is an individual’s group size. Playback experiments testing the ability of individuals to assess the size of intruding groups have identified group size as a factor affecting individual response to playback, with individuals more likely to approach the speaker following playback as their group size increased (McComb et al. 1994; Benson‑Amram et al. 2011). Furthermore, group size has been found to be positively associated with cognitive performance in several species (Johnson-Ulrich and Holekamp 2020; Speechley et al. 2024a, b; Triki et al. 2024), including the Western Australian magpie (Ashton et al. 2018a), supporting the social intelligence hypothesis (SIH) which states that group living can create challenges that select for advanced cognitive abilities (Dunbar 1998; Ashton et al. 2018b). Another factor that may be a source of individual variation in performance on quantity discrimination tasks is sex. Males of many species participate more in intergroup interactions than females (Perry 1996; Benadi et al. 2008; McDonald et al. 2012). Where this is the case, the quantity discrimination abilities of males may be under stronger selection than for females, as males benefit more from being able to assess the quantity of intruding individuals during an intergroup interaction. In addition, males may benefit more from the ability to judge the quantity of conspecific males present during the breeding season. For example, extra-group paternity in Western Australian magpies is extremely high (> 80%; Hughes et al. 2003), and hence males may benefit from the ability to assess the number of rival males in unknown groups when on extra-group forays for mating opportunities.

In this study, we presented wild Western Australian magpies with a spontaneous quantity discrimination task to (i) determine whether this species possesses quantity discrimination abilities, and (ii) investigate the factors affecting individual variation in spontaneous quantity discrimination performance. We predicted that individuals from larger groups will perform better on the task than individuals from smaller groups, and that males will perform better than females.

## Methodology

### Study species and site

The Western Australian magpie (hereafter magpie) is a subspecies of Australian magpie that inhabits the south-west of Western Australia (Johnstone 2004). The magpie is a large (250–400 g) passerine that lives year-round in stable cooperatively breeding groups ranging from three to 12 individuals (Pike et al. 2019). Magpies achieve full adult plumage at approximately three years of age, at which point they are sexually dichromatic, with males possessing a white back and females possessing a scalloped black and white back (Johnstone 2004). Magpies are highly territorial, engaging in vocal and visual territory defence displays and participating in frequent disputes with other groups (Pike et al. 2019; Blackburn et al. 2022b).

This study included 42 adult magpies (20 males and 22 females) from 11 groups located in Crawley (31.9752° S, 115.8213° E) and Guildford (1.8994° S, 115.9717° E), Perth. Groups ranged in size from 3 to 15 individuals (mean ± SE = 7.18 ± 1.17 individuals). Information on the sex and group size distribution of magpies tested in this study is available in the Supplementary Materials (Table S1). All groups were situated in urban parks or grasslands. The study population has been monitored since 2013 and is habituated to the presence of humans, enabling detailed behavioural observations and the presentation of cognitive tasks (Ashton et al. 2018a). Individuals within each group are identifiable by unique colour-ring combinations or distinct physical features such as plumage patterns or scarring (Ashton et al. 2018a).

### Experimental design

**Fig. 1:**
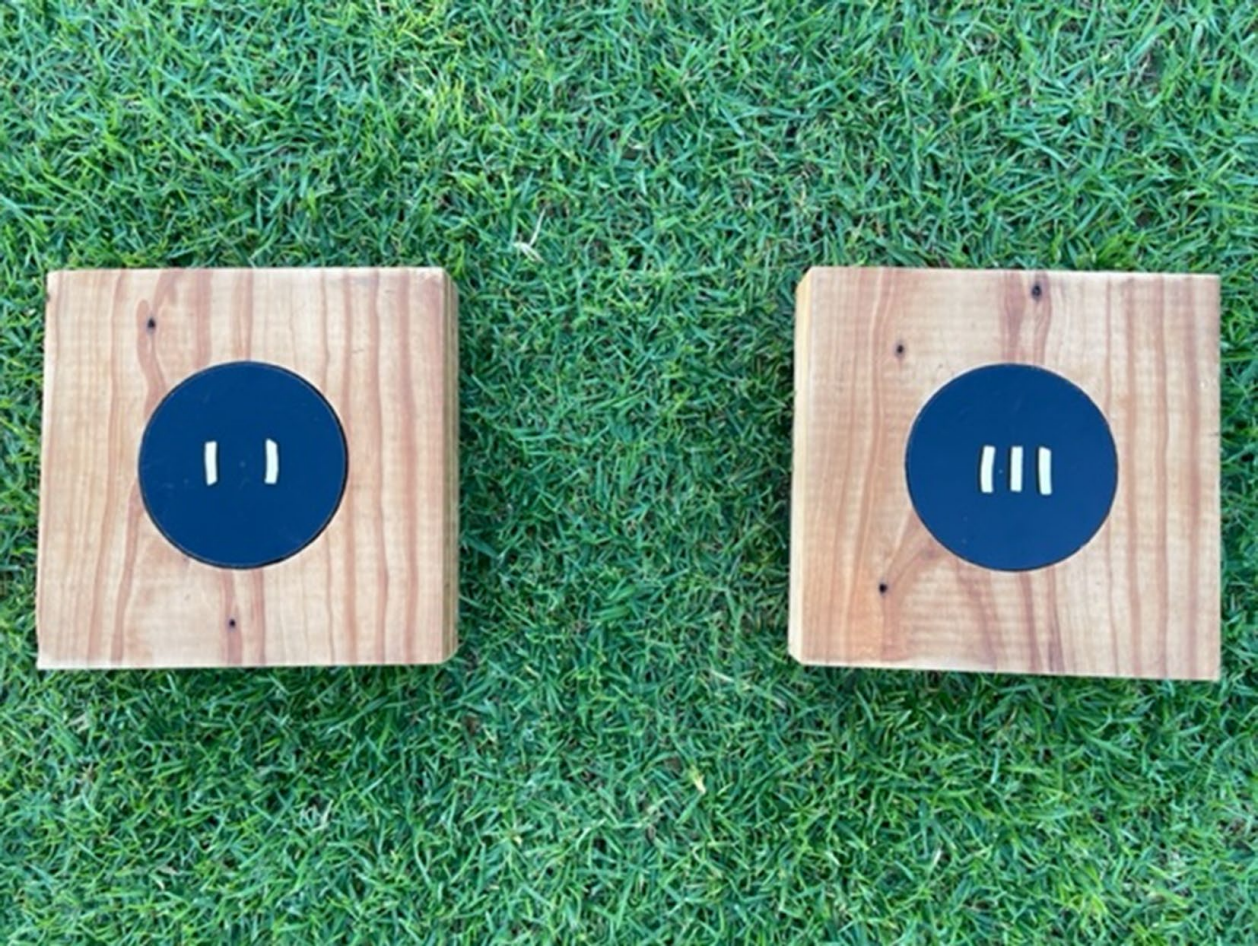
The spontaneous quantity discrimination task used to test quantity discrimination in Western Australian magpies, displaying the 2 vs. 3 ratio

The spontaneous quantity discrimination task consisted of two wooden boards (20 cm x 20 cm x 5.5 cm), each with a well (10 cm diameter, 3 cm deep) in the centre covered by a black plastic lid (Fig. 1). Equal amounts of mozzarella cheese were placed into the well of each wooden board, to attempt to control for the use of olfactory cues which may be present because of the different cheese ratios placed on top of the lids. It is important to note the possibility that the plastic lids covering the wells may have prevented magpies from smelling the cheese, and hence this may not have completely controlled for olfactory cues as intended. Each lid was fitted into place by the experimenter and was immovable by the magpies. Testing followed the protocol of Blackburn et al. (2024), with 2.5 cm strands of mozzarella cheese placed on each lid in the following ratios: 2 vs. 2 (control), 2 vs. 3 (ratio of 0.66̅), 2 vs. 4 (ratio of 0.5) or 2 vs. 5 (ratio of 0.4). Mozzarella cheese was used because it is a preferred food reward previously used during cognitive testing in this study population (Ashton et al. 2018a; Blackburn et al. 2024). Ratios including the number one were excluded from the design to eliminate the likelihood of individuals simply discriminating one from many. The 2 vs. 2 ratio was presented as a control trial to test for the presence of any side bias. The two lids were identically marked across the centre with six small lines, spaced 1 cm apart, to control for horizontal space when placing cheese strands (as per Blackburn et al. 2024). Dark blue was chosen as a marker for the lines because this colour was similar to the lid colour and thus was unlikely to act as a colour cue for the magpies.

Focal birds were selected randomly from each group while aiming to ensure an even representation of males and females, and birds from groups of different sizes for robustness (Table S1). Focal individuals completed four trials per day (one trial of each ratio) for a period of 15 days, resulting in 60 trials per bird. All 15 testing days for each individual were completed within four weeks to limit any potential effect of seasonality on trial results. The four trials were presented in a randomised order each day, with the side holding the greater food reward also randomised between trials. All individuals tested on the same day were presented with the same randomised order. All trials were recorded using a GoPro, allowing us to quantify neophobia (time taken to approach the task from one metre to the first contact with a board), time spent interacting with the task (time from first contact to moving away) and time between trials. Time of day, temperature, and weather were recorded at the beginning of each trial from the Bureau of Meteorology application (from the nearest weather station; 4 km away from Guildford, and 7 km from Crawley). Group identity, group size (including fledglings and juveniles), and sex were recorded for each bird. Each bird was tested in isolation (not within 10 m of other group members, *sensu* Ashton et al. 2018a) to minimise the effects of social interference and social learning on task performance. The focal bird was isolated either by waiting until they had moved away from other group members (magpie often forage > 10 m apart from group members; Ashton et al. 2018a) or by a second experimenter drawing other group members aways from the focal individual with mozzarella cheese. The presence of vegetation and trees in the study area was also used to visually isolate focal birds from group members during testing (*sensu* Blackburn et al. 2022a). If another magpie came within 10 m of the focal bird during the trial, that trial was terminated and not restarted until the focal bird was again socially isolated.

For each trial, cheese strands for the selected ratio were placed uniformly on each lid. The boards were set up out of sight of the focal bird. The boards were then placed approximately two meters in front of the focal individual (while the focal bird was facing the experimenter) and approximately 30 cm apart, oriented in the same direction, with the bird equidistant between the two boards. The bird was then allowed to approach the boards and select a food reward from one side: this was noted as the focal bird’s choice. Individuals could only consume the food reward from the board they selected, meaning if they selected the smaller food reward they received less food. Once the full amount of food from the selected board had been consumed, both boards were removed. The boards were then reset for the next trial out of the bird’s sight. Once reset, the boards were presented immediately, however if the bird had taken cheese away from the previous trial to consume, the second trial was not presented until the bird had finished eating. The focal individual completed all four trials before the next individual in the group was tested. All trials were conducted in the morning and testing was conducted as soon as possible following sunrise to reduce the possibility of pre-testing satiation. Where possible, individuals were tested at similar times of day to control for any differences related to the time of day.

### Statistical analysis

To determine if magpies possessed spontaneous quantity discrimination abilities and to investigate the factors influencing individual variation in this ability, we performed statistical analyses using R Studio version 4.1.1 (RStudio Team 2022). To determine whether there was a left- or right-side bias, a binomial test was conducted on the 2 vs. 2 control trials for all individuals to see whether one side was chosen more often than the other. To firstly determine the presence of quantity discrimination abilities (prior to analysis of factors affecting performance), a binomial test was conducted on performance on each of the other ratios (2 vs. 3, 2 vs. 4, and 2 vs. 5) to determine if the larger food reward was chosen non-randomly for each ratio.

Using the lme4 package (Bates et al. 2015), we ran generalised linear mixed-effects models (GLMMs) with a binomial distribution and logit link function to determine the causes of individual variation in cognitive performance. Group and bird identity were included as random terms in all models (variance and SD explained by random terms is shown in Table S2). The binomial response term was pass (i.e. individual chose the larger food reward) or fail (i.e. individual did not choose the larger food reward). Explanatory terms included were the ratio presented (2 vs. 3, 2 vs. 4, or 2 vs. 5), sex, temperature, trial number, weather, group size, neophobia, side chosen (left or right), and time spent interacting with the task (seconds). Interactions between the ratio presented and individual attributes (group size and sex) were investigated, as the effect of these attributes may differ across the ratios tested (e.g. group size may more strongly affect the ability of birds to discriminate between more disparate quantities such as 2 vs. 5, as this might be more beneficial in contest and foraging scenarios). Group size was a continuous measure of the total number of individuals in the focal birds’ social group, including fledglings and juveniles, and was determined using the life history database, which contained information updated weekly on group composition. Temperature (^o^C) refers to the temperature recorded at the beginning of each trial. Trial number (1–15) was included as an explanatory term to investigate the possibility that magpies were improving in their performance over the course of testing. Weather was noted at the beginning of each trial and described as either clear, cloudy, or raining. Neophobia was quantified as the time taken (s) by each bird to approach and touch their selected board once they came within one meter of the boards. Time interacting refers to the length of time (s) spent interacting with the task in each trial, beginning from the moment the test subject first made contact with the board and ending when the subject either moved away or the board was removed. Time interacting was found to correlate with food ratio, as a greater food amount takes a longer time to eat, therefore it was removed from the analysis.

Five individuals were identified as having a side-bias (chose the left or right side in ≥ 12 out of the 15 2 vs. 2 trials, representing a significant deviation from binomial probability). Therefore, a second analysis was run excluding these individuals (*N* = 1665 trials on 37 magpies from 11 groups). Results did not differ between this and the main analysis, therefore we retained the analysis including all individuals in the main text and present the analysis excluding the five individuals with a side bias in the Supplementary Materials (Table S5, Fig. S1 and S2).

### Model selection

Akaike’s Information Criterion values corrected for small sample sizes (AICc) were used to determine which explanatory terms were the best predictors of data patterns. A range of models, each representing a biological hypothesis, were tested. A basic intercept model including only the intercept and random terms was included. Only parameters whose confidence intervals did not intersect zero when tested alone were included in further additive models, however terms whose confidence intervals did intersect zero when tested alone could still be considered in interactions. The model with the lowest AICc value was considered the most parsimonious model, provided the confidence intervals of the predictor terms’ effect sizes did not intersect zero. All models that were within two AICc of the top model were included in the top model set. Predictor terms in models were considered significant if their parameter 95% confidence intervals did not intersect zero (Grueber et al. 2011; Symonds and Moussalli 2011).

### Post-hoc comparisons

We used the *emtrends* function within the emmeans package (Lenth 2019) in R to obtain contrasts between the different ratios presented and group size. *P*– values were adjusted for multiple comparisons via the Tukey method.

## Results

A total of 42 magpies were tested 15 times on each of the four ratios (2 vs. 2, 2 vs. 3, 2 vs. 4 and 2 vs. 5), resulting in 630 individual trials for each ratio (*N* = 2520 trials in total). A binomial test on the 2 vs. 2 trials revealed no side bias at the population level (binomial test: *N* = 42, *P* = 0.968). The 2 vs. 2 trials were excluded from further analyses (sample size for further analyses was therefore *N* = 1890 trials on 42 magpies).

### Spontaneous quantity discrimination performance

**Fig. 2:**
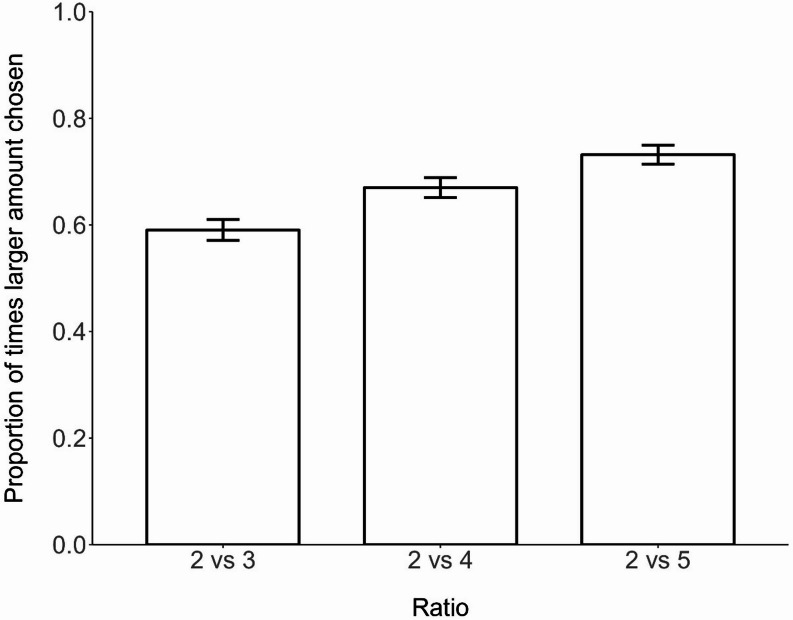
Mean proportion of times focal magpies selected the larger amount of food in the spontaneous quantity discrimination task. Error bars represent standard error. *N* = 1890 trials completed by 42 magpies from 11 groups

Binomial tests confirmed that the number of times individuals selected the larger food reward was significantly higher than chance in the 2 vs. 3 (*N* = 630 trials on 42 magpies, *P* < 0.001; larger amount was chosen in 59% of trials), 2 vs. 4 (*N* = 630 trials on 42 magpies, *P* < 0.001; larger amount was chosen in 67% of trials), and 2 vs. 5 (*N* = 630 trials on 42 magpies, *P* < 0.001; larger amount was chosen in 73% of trials) ratios, establishing the capacity of magpies to discriminate quantities at every ratio tested. The proportion of trials in which the larger food reward was selected increased as the ratio decreased from 2 vs. 3 (ratio of 0.66̅), to 2 vs. 4 (ratio of 0.5) (GLMM: z = 2.96, *N* = 42, *P* = 0.003) to 2 vs. 5 (ratio of 0.4) (GLMM: z = 5.34, *N* = 42, *P* < 0.001) (Fig. 2).

### Factors affecting individual variation in performance

**Fig. 3:**
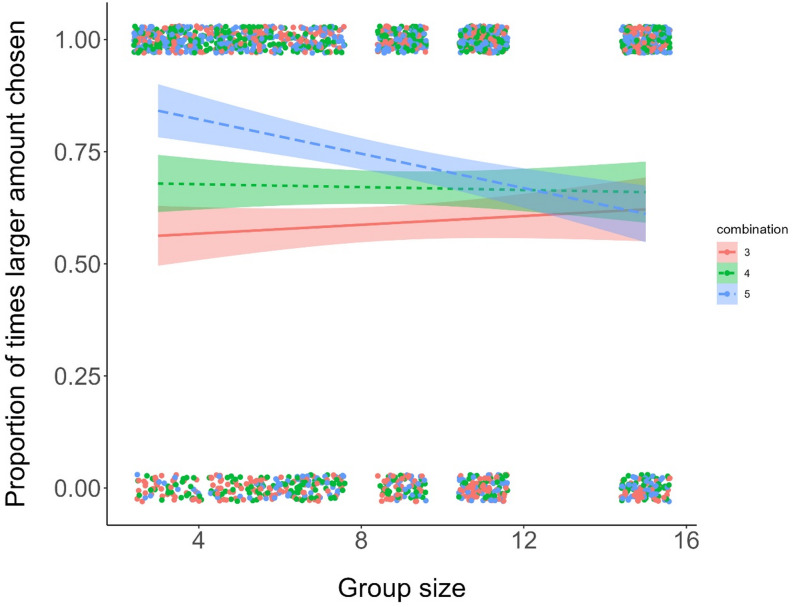
Proportion of times magpies selected the larger quantity of food in the spontaneous quantity discrimination task in relation to their group size. Red = 2 vs. 3 ratio, green = 2 vs. 4 ratio, and blue = 2 vs. 5 ratio. Points represent pass or fail of each trial and are jittered for clarity; shaded areas represent 95% confidence intervals. *N* = 1890 trials completed by 42 magpies from 11 groups

Group size alone did not significantly affect the ability of individuals to choose the larger quantity of food in the spontaneous quantity discrimination task (95% CI = -0.233, 0.271; Table 1; Table S3). However, the interaction between group size and ratio presented was significant, with magpies from smaller groups choosing the larger food reward in the 2 vs. 5 trials significantly more than magpies from larger groups (Table 1; Fig. 3). Post-hoc analyses support these results (Table S4). We found no effect of sex on spontaneous quantity discrimination performance (Table S3). Trial number also did not affect the probability of magpies choosing the larger food reward, indicating that individuals do not improve in their performance over time (Table S3).

**Table 1:** Top model set of terms affecting performance on the spontaneous quantity discrimination task. All models included group and bird ID as random terms. The top model set includes terms within 2 AICc of the best model. Coefficient estimates ± s.e. and 95% confidence intervals (CI) are given below the top model set. For the full set of candidate terms tested, see Table S3. Output is based on a GLMM analysis with a binomial distribution and a logit link function from 1,890 trials completed by 42 magpies from 11 groups.

Top models	AICc	∆AICc
Group size * Ratio	2361.35	0.00
*Null model*	2400.87	39.52
**Parameter**	**Estimate ± S.E.**	**95% CI**
Group size	0.019 ± 0.129	-0.233; 0.271
Ratio		
2 vs. 3	-	-
2 vs. 4	0.352 ± 0.119	0.119; 0.585
2 vs. 5	0.700 ± 0.125	0.454; 0.945
Group size * Ratio		
Group size * 2 vs. 3	-	-
Group size * 2 vs. 4	-0.121 ± 0.121	-0.358; 0.117
Group size * 2 vs. 5	-0.525 ± 0.128	-0.775; -0.274

## Discussion

We found that Western Australian magpies were able to discriminate between different quantities of a food reward, with magpies choosing the larger quantity of food more often than expected by chance in all ratios presented. Furthermore, our results identified group size as a factor affecting individual variation in performance on a spontaneous quantity discrimination task, with individuals from smaller groups performing significantly better than individuals from larger groups when presented with the 2 vs. 5 ratio.

Magpies performed with increasing aptitude to select the larger food reward as the distance between the quantities presented increased (i.e. as the ratio decreased). Such an increase in discrimination accuracy as ratio decreases has been observed in other species (Ward and Smuts 2007; Hunt et al. 2008; Jones and Brannon 2012; Stancher et al. 2015; Tomonaga et al. 2023). For example, prosimian primates selected the larger reward in a 2 vs. 3 ratio (ratio of 0.66̅ ) in only 40% of trials, however when the ratio decreased to 1 vs. 3 (ratio of 0.33̅), the larger amount was selected in 80% of trials (Jones and Brannon 2012). Such findings support the hypothesis that animals employ the approximate number system (ANS) when discriminating between quantities. The ratio effect of ANS implies that performance is constricted not by absolute values but by the ratio between quantities (Bosshard et al. 2022). From an ecological point of view, a ratio effect seems efficient (Beran et al. 2015; Bosshard et al. 2022); being able to differentiate between a food source with 12 items and a food source with two items, for example, has a clear nutritional benefit (assuming similar size between items), whereas choosing between a food source with 12 items and a food source with 11 items does not provide an equivalent benefit. Future studies should conduct additional testing incorporating more quantity combinations that represent both the same and different ratios to (i) confirm the use of the ANS in magpies and investigate whether the OFS exists in this species and is employed for smaller quantities, and (ii) further investigate the extent of quantity discrimination abilities in this species, as they likely exceed those tested here.

The interaction between group size and ratio significantly affected the spontaneous quantity discrimination performance of magpies, with individuals from smaller groups performing better on the 2 vs. 5 ratio (but not the 2 vs. 3 or 2 vs. 4 ratios) compared to individuals from larger groups. Such a finding contrasts with previous work on this species that has consistently found a positive relationship between group size and cognitive performance across several other cognitive tasks (Ashton et al. 2018a; Blackburn et al. 2022a; Speechley et al. 2024a, b). The differing influence of group size on different cognitive traits suggests that these may be under selection from different pressures. Smaller groups are generally more likely to experience increased costs as a result of intergroup conflict compared to larger groups. For example, smaller groups may be more likely to lose contests, be displaced, or go extinct compared to larger groups (Radford and du Plessis 2004; Keynan and Ridley 2016; Dyble et al. 2019; Majolo et al. 2020; Ridley et al. 2022). Individuals in smaller groups may also be more likely to be injured in an intergroup conflict compared to individuals in larger groups, as there are less individuals in smaller groups to share the costs of intergroup conflict (Hamilton 1971; Wrangham 1999; Pavez‑Fox et al. 2023). For these reasons, individuals from smaller groups may benefit more than those from larger groups by being able to perceive and quantify the size of conspecific groups.

An alternative explanation for the group size effect identified in this study is related to the many-eyes hypothesis, which states that as the size of a group of foraging animals increases, the number of eyes searching the area for threats increases, therefore individuals can dedicate less time to vigilance and more time to foraging and other activities (Caraco et al. 1980; Lima 1995). Animals in larger groups may therefore afford to be less selective when it comes to choosing the greatest food reward, as they are able to forage for longer to obtain their resource requirements. Conversely, individuals from smaller groups, who must allocate more time to vigilance and subsequently less time to foraging (Caraco et al. 1980; Beauchamp et al. 2021) may gain a greater advantage in being able to make accurate assessments regarding the availability and quantity of food in foraging patches. Interestingly, the relationship between magpie cognitive performance and group size was only significant on the 2 vs. 5 ratio, and not on the 2 vs. 3, or 2 vs. 4 ratios. It may be the case that being able to discriminate between five and two (both in terms of food items and competitors) is more beneficial than being able to discriminate between three and two, therefore selection may be stronger for this more broad-scale and ratio-dependent quantity discrimination, particularly in smaller groups for whom this ability is likely more useful.

This study is one of the first to investigate the causes of individual variation in quantity discrimination abilities in a population of wild animals. We first identified that magpies possess quantity discrimination abilities and demonstrate performance consistent with the use of the ANS of discrimination. We then provided evidence that individuals from smaller groups are better able to discriminate between food rewards presented in a 2 vs. 5 ratio compared to individuals from larger groups. This is consistent with the idea that the adverse consequences of intergroup conflict are greater for individuals in smaller groups, and hence selection for quantity discrimination abilities is stronger for these individuals. Whether group size influences the development of cognitive phenotypes during early life (as has been seen with other cognitive traits in this species; Ashton et al., 2018a, 2018b), or later life requires further investigation and repeated cognitive testing of juvenile magpies over time. Nevertheless, the results of this study enhance our understanding of how individual differences in the quantity discrimination abilities of social species arise and highlight the importance of considering intraspecific variation when investigating the cognitive ecology of this trait.

## Electronic supplementary material

Below is the link to the electronic supplementary material.

Supplementary Material 1

## Data Availability

Data are available from the Figshare data repository, 10.6084/m9.figshare.28252883.
