# Supplementary material for: Group size affects spontaneous quantity discrimination performance in wild Western Australian magpies (Gymnorhina tibicen dorsalis)
Source: Anim Cogn. 2025 May 26;28(1):41. doi: 10.1007/s10071-025-01963-0 (PMC12106560; doi:10.1007/s10071-025-01963-0)
Supplement: Supplementary file 1 — Supplementary Material 1 [file 10071_2025_1963_MOESM1_ESM.docx]

**Group size affects spontaneous quantity discrimination performance in wild Western Australian magpies (***Gymnorhina tibicen dorsalis***)**

**Supplementary material**

**Table S1.** Information on individuals tested on the spontaneous quantity discrimination task.

| **Group size** | **Number of birds tested** |
| --- | --- |
| 3 | 5 |
| 4 | 3 |
| 5 | 4 |
| 6 | 3 |
| 7 | 4 |
| 9 | 6 |
| 11 | 9 |
| 15 | 8 |
| **Sex** | **Number of birds tested** |
| Male | 20 |
| Female | 22 |

**Table S2.** Variance and standard deviation (SD) explained by random terms (bird ID and group ID) in the null (intercept only) model and top model for terms affecting spontaneous quantity discrimination performance. *N =* 1890 trials completed by 42 magpies from 11 groups.

| **Model** | **Random term** | **Variance** | **SD** | **Random term** | **Variance** | **SD** |
| --- | --- | --- | --- | --- | --- | --- |
| Null model (intercept only) | Bird ID | 0.071 | 0.266 | Group ID | 0.074 | 0.272 |
| Top model (group size * ratio) | Bird ID | 0.077 | 0.277 | Group ID | 0.066 | 0.257 |

**Table S3.** Full model set of terms affecting performance on the spontaneous quantity discrimination task. All models included group and bird ID as random terms. Corrected Akaike information criterion (AICc) and ∆AICc are provided for each candidate model. Only within 2 AICc of the top model, and with predictors whose 95% confidence intervals did not intersect zero were included in the top model set and are highlighted in bold. *N* = 1890 trials completed by 42 magpies from 11 groups.

| **Model** | **AICc** | **ΔAICc** |
| --- | --- | --- |
| **Group size * ratio** | **2361.35** | **0** |
| Sex + ratio | 2374.89 | 13.54 |
| Group size + ratio | 2375.47 | 14.12 |
| Ratio | 2375.77 | 14.42 |
| Sex * ratio | 2382.20 | 20.85 |
| Side chosen | 2386.57 | 25.22 |
| Group size * sex | 2397.63 | 36.28 |
| Sex | 2400.34 | 38.99 |
| Group size | 2400.52 | 39.17 |
| *Null model* | 2400.87 | 39.52 |
| Temperature | 2401.88 | 40.53 |
| Neophobia | 2402.73 | 41.38 |
| Trial number | 2402.96 | 41.61 |
| Weather | 2403.10 | 41.75 |

| **Contrast** | **Estimate** | **SE** | **z-ratio** | ***P*-value** |
| --- | --- | --- | --- | --- |
| 2 vs 3 ratio performance * Group size | 0.019 | 0.129 | 0.148 | 0.883 |
| 2 vs 4 ratio performance * Group size | -0.102 | 0.132 | -0.772 | 0.440 |
| 2 vs 5 ratio performance * Group size | -0.505 | 0.139 | -3.623 | 0.001 |

**Table S4.** Post-hoc analyses of spontaneous quantity discrimination performance by total group size. *N =* 1890 trials completed by 42 magpies from 11 groups.

**Analysis of factors affecting performance on the spontaneous quantity discrimination task on a subset of data excluding five individuals identified as having a side bias:**

**Table S5**. Full model set of terms affecting performance on the spontaneous quantity discrimination task, using a subset of data which excluded five individuals that were identified as having a side bias. All models included group and bird ID as random terms. Corrected Akaike information criterion (AICc) and ∆AICc are provided for each candidate model. Only within 2 AICc of the top model, and with predictors whose 95% confidence intervals did not intersect zero were included in the top model set and are highlighted in bold. *N* = 1665 trials completed by 37 magpies from 11 groups.

| **Model** | **AICc** | **ΔAICc** |
| --- | --- | --- |
| **Group size * ratio** | **2068.00** | **0** |
| Ratio | 2079.15 | 11.15 |
| Sex * ratio | 2085.30 | 17.30 |
| Side chosen | 2093.22 | 25.22 |
| Group size * sex | 2103.03 | 35.03 |
| Group size | 2105.30 | 37.30 |
| *Null model* | 2105.64 | 37.64 |
| Temperature | 2105.76 | 37.76 |
| Sex | 2105.81 | 37.81 |
| Trial number | 2107.28 | 39.28 |
| Neophobia | 2107.58 | 39.58 |
| Weather | 2107.77 | 39.77 |


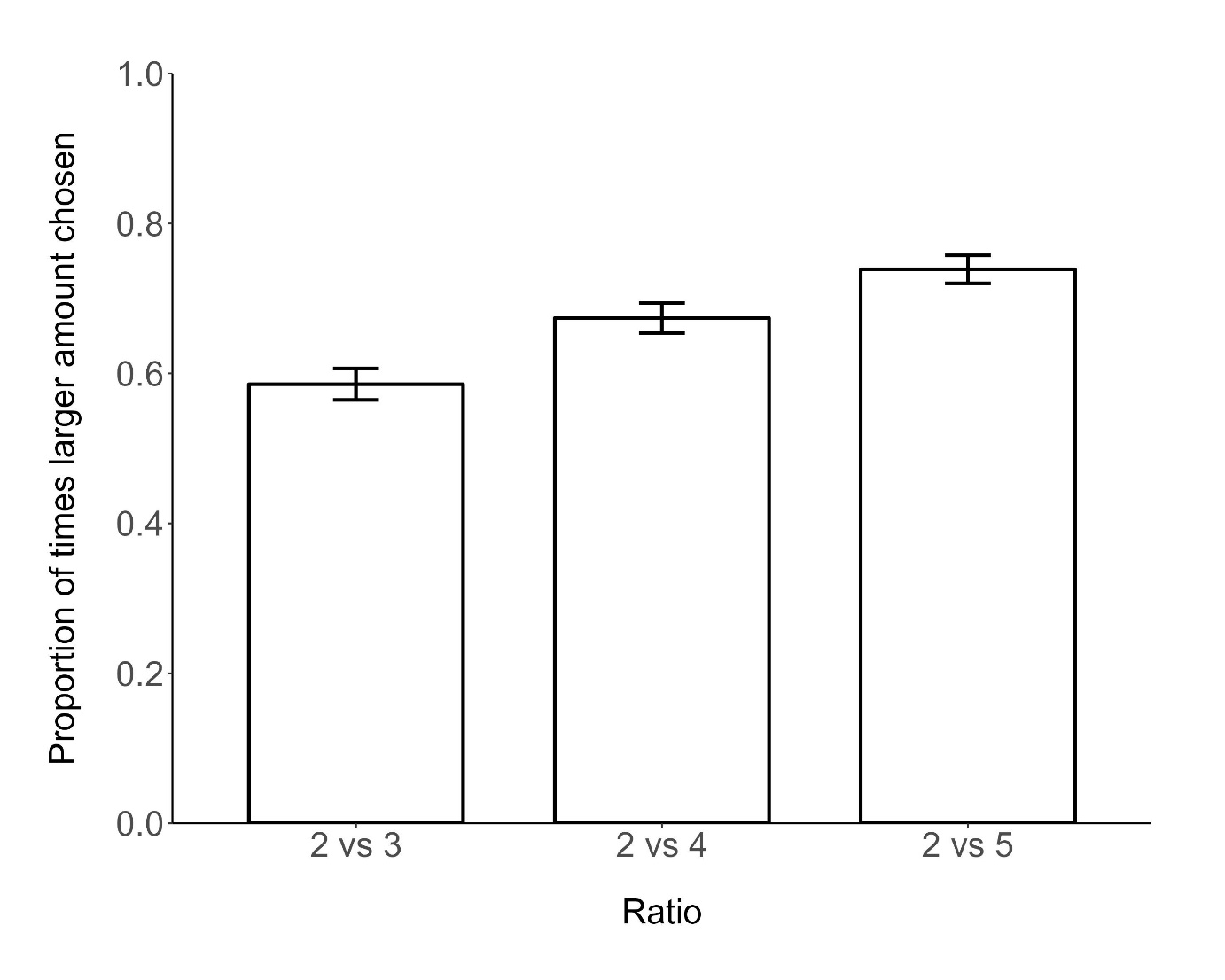
**Fig. S1** Mean proportion of times focal magpies selected the larger quantity of food in the spontaneous quantity discrimination task, using a subset of data which excluded five individuals that were identified as having a side bias. Error bars represent standard error. *N* = 1665 trials completed by 37 magpies from 11 groups

**
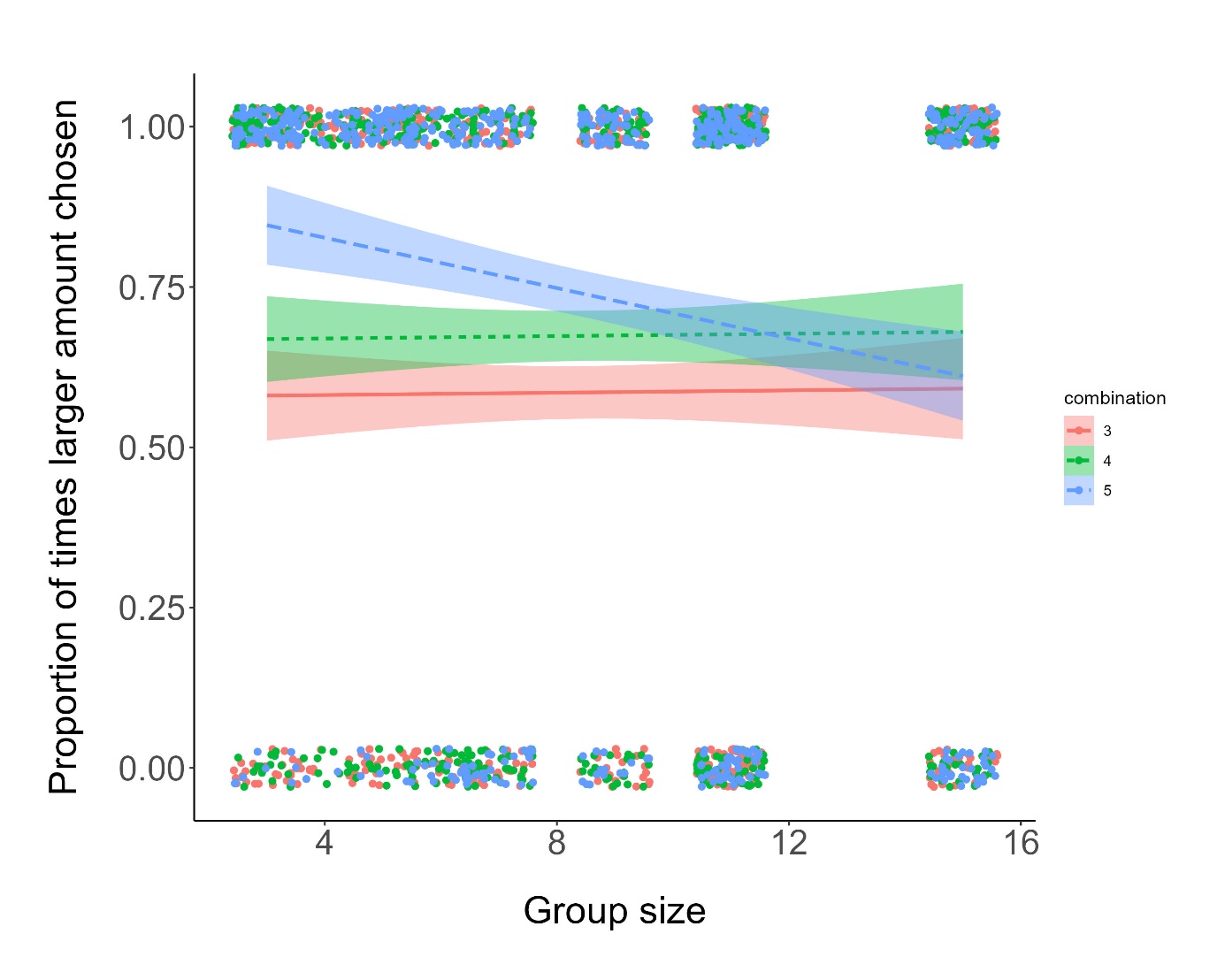
Fig. S2** Proportion of times magpies selected the larger quantity of food in the spontaneous quantity discrimination task in relation to their group size, using a subset of data which excluded five individuals that were identified as having a side bias. Red = 2 vs 3 ratio, green = 2 vs 4 ratio, and blue = 2 vs 5 ratio. Points represent pass or fail of each trial and are jittered for clarity; shaded areas represent 95% confidence intervals. *N* = 1665 trials completed by 37 magpies from 11 groups
